# Setting up a clinical psychology service for reception department with consultative care

**DOI:** 10.1192/j.eurpsy.2021.1039

**Published:** 2021-08-13

**Authors:** N. Semenova, S. Shport

**Affiliations:** Psychiatry, Moscow Research Institute of Psychiatry – a branch of V.Serbsky National Medical Research Centre for Psychiatry and Narcology, Moscow, Russia, Moscow, Russian Federation

**Keywords:** Reception Department, Consultees, Brief intervention service, Clinical psychology

## Abstract

**Introduction:**

There is a plenty of literature on providing psychological services for psychiatry inpatients and outpatients. Seeing a psychiatrist for the first time can be stressful.

**Objectives:**

This paper will identify challenges in setting up a clinical psychology service for consultees seen in Reception Department with consultative and primary specialized health care. ‘Clinical psychology service’ is a project in Moscow Research Institute of Psychiatry providing services within the Reception Department setting. A need to address mental ill health issues (getting a diagnosis and treatment plan) within the broader psychosocial needs of consultees has been identified.

**Methods:**

Clinical psychology established a brief intervention ‘service’ for consultees. Issues of establishing trust within this population suggested the need to provide a ‘named’ male or female psychologist. The psychologist accompanied psychiatric consultants’ recommendations to familiarize a consultee of the availability of the service. Evaluation of the service, including uptake, client satisfaction, and outcome, is ongoing using quantitative and qualitative methods. Data is presented on key themes identified in providing psychological services to consultees.

**Results:**

Key themes identified included: 1. Service development: establishing trust, ensuring confidentiality, close between consultants working, flexibility, crisis management, safety, establish links with other agencies; 2. Complex psychological and social needs: mental health issues, trauma, substance misuse, domestic and sexual violence.

**Conclusions:**

Consultees present with a range of complex psychosocial needs. While this population may have reservations about accessing ‘standard’ mental health services, a flexible psychology service working in close liaison with psychiatric consultants may be effective in addressing these needs.

